# An iceberg I can’t handle: a qualitative inquiry on perceptions towards paediatric rheumatology among healthcare workers in Kenya

**DOI:** 10.1186/s12969-023-00790-2

**Published:** 2023-01-21

**Authors:** Angela Migowa, Sasha Bernatsky, Anthony Ngugi, Helen E. Foster, Peter Muriuki, Adelaide Lusambili, Stanley Luchters

**Affiliations:** 1 International Centre for Reproductive Health, Department of Public Health and Primary Care, Ghent University, Ghent, Belgium; 2 Department of Paediatrics and Child Health, Aga Khan University, Medical College East Africa, Nairobi, Kenya; 3 Department of Medicine (Division of Rheumatology and Epidemiology) McGill University Health Centre (MUCH), Montreal, Canada; 4 Department of Population Health, Aga Khan University East Africa, Nairobi, Kenya; 5 Population and Health Institute, Newcastle University, Newcastle, UK; 6 African Population and Health Research Centre, Nairobi, Kenya; 7 Centre for Sexual Health and HIV AIDS Research (CeSHHAR), Harare, Zimbabwe; 8 Liverpool School of Tropical Medicine (LSTM), Liverpool, UK

**Keywords:** Paediatric rheumatology, Health care workers, Knowledge, Attitudes, Practices, Kenya, Sub-Saharan Africa

## Abstract

**Background:**

Delay in diagnosis and access to specialist care is a major problem for many children and young people with rheumatic disease in sub-Saharan Africa. Most children with symptoms of rheumatic disease present to non-specialists for care. There is an urgent need to understand and scale-up paediatric rheumatology knowledge and skills amongst non-specialist healthcare workers to promote early diagnosis, prompt referral, and management.

**Purpose:**

We evaluated the knowledge, attitudes and practices towards diagnosis and care of paediatric rheumatology patients among health care workers in Kenya.

**Methods:**

We conducted 12 focus group discussions with clinical officers (third-tier community health workers) nurses, general practitioners and paediatricians across 6 regions in Kenya. Interviews were conducted on zoom, audio-recorded, transcribed, and analysed using NVIVO software.

**Results:**

A total of 68 individuals participated; 11 clinical officers, 12 nurses, 10 general practitioners, 27 paediatricians and 7 others. Most (*n* = 53) were female, and the median age was 36 years (range 31–40 years). Fifty per cent of the participants (34 of 68) worked in public health facilities. Our study revealed gaps in knowledge of paediatric rheumatology amongst healthcare workers which contributes to delayed diagnosis and poor management. Healthcare workers reported both positive and negative attitudes towards diagnosis and care of paediatric rheumatology patients. Perceived complexity and lack of knowledge in diagnosis, management and lack of health system clinical pathways made all cadres of healthcare workers feel helpless, frustrated, inadequate and incompetent to manage paediatric rheumatology patients. Positive attitudes arose from a perceived feeling that paediatric rheumatology patients pose unique challenges and learning opportunities.

**Conclusion:**

There is an urgent need to educate healthcare workers and improve health systems to optimize clinical care for paediatric rheumatology patients.

**Supplementary Information:**

The online version contains supplementary material available at 10.1186/s12969-023-00790-2.

## Introduction

Paediatric rheumatic diseases such as Juvenile Idiopathic Arthritis (JIA), are painful chronic potentially very disabling conditions associated with significant mortality, morbidity and reduced quality of life [1]. Rheumatic diseases create a huge burden in high-, middle-, and low- income countries [2]. In Europe for example, rheumatic diseases are estimated to cost more than 200 billion euros per year and are considered the most expensive diseases for healthcare systems [3]. Globally, about 6–7 million children are affected by rheumatic diseases, which causes significant mortality and morbidity among children [1]. It is estimated that approximately 30% of these children live in Africa [1, 4–6].

Africa has 25% of the world’s rheumatic disease burden but only 2% of the world’s human resources for clinical care [7]. The paediatric rheumatology (PR) burden in Africa is likely underestimated given the paucity of rheumatologists among other factors [8]. Inadequate education, socio-cultural beliefs and limited access to care aggravate the situation [9–12].

A central mission for the paediatric rheumatology workforce is to provide children with healthcare access and optimum clinical outcomes [9]. The current supply of rheumatologists is insufficient in many countries [13]. A review by Henrickson revealed that the proportion of paediatric rheumatologists across the globe is 0.08 PR/million in Africa, 0.04 PR /million in Asia, 0.2 PR/million in Latin America, 2.4 PR/million in Europe, 3–4 PR/ million in North America, 6.3 PR / million in Oceania and 6.7 PR/million in Middle East [9].

There are only 10 paediatric rheumatology centres across the 54 countries in Africa [14]. In other resource-constrained countries such as India, there are 15 paediatric rheumatologists for 444 million children [15, 16]. In contrast, the United States is estimated to have 266 paediatric rheumatologists for a population of 67 million [17]. The recommended ratio of paediatric rheumatologists in each population ranges from 1:300,000 to 1:500,000 [18].

As per the most recent national census by the Kenya National Bureau of Statistics (KNBS) in 2019, Kenya’s population was estimated at 47,564,296 [19]. The paediatric population in Kenya is estimated to be about 53% approximately 24,920,161 million [20, 21]. In order to improve healthcare efficiency, Kenya has undergone a series of reforms that culminated in devolution of healthcare service provision from the national level to the new 47 county local level governments [22]. Under the devolved system, the national government is responsible for health policy, national teaching and referral health facilities, while the county governments are entrusted with all functions related to health service delivery up to the county referral hospital [22].

The Ministry of Health is still the major financier and provider of health-care services in Kenya, controlling about 52% of the health facilities with an expenditure on health per capita of $51 US [23]. The other 48% is distributed among local county governments, private institutions, mission organisations and non-governmental organizations [23]. In the fiscal year 2018/2019, the health sector, including the national and county levels, shared only 5.4% of the national budget which was about one-fifth of that for education [22]. Expanding fiscal space for health is critical to ensure that Kenya moves towards universal health coverage (UHC) to achieve sustainable development goals by 2030 [22].

The public universities in Kenya are historically funded by the Government which supports both undergraduate and postgraduate university education for Kenyans [23]. Due to the increasing demand for a university education, the Government alone is not in a position to fully finance university education [23]. In Kenya, the increased demand for university education and the inability to meet these demands has led to the introduction of a system of training self-sponsored medical students alongside Government-subsidised students [23].

The ratio of doctors to population is estimated at 17 per 100,000 [23]. Due to the decreasing number of trained doctors and their continuous migration, Kenya suffers a chronic shortage of doctors, particularly in the public sector [23]. Consequently, the bulk of healthcare services in Kenya is provided by para-medicals such as clinical officers (COs) [23]. Clinical officers (COs) are non-physician clinicians who have received less training, have a more restricted scope of practice than professionals, and are accredited by their countries’ licensing bodies [22].

The Kenya Medical Training College (KMTC) has a network of 71 campuses, strategically located across the country in 45 out of 47 counties [24]. It offers pre-service nursing/midwifery and clinical officer training across the country [24] in various health care disciplines [25].

Currently there are only 2 practicing paediatric rheumatologists in Kenya [14]. Since Africa is currently estimated to have 15 practicing paediatric rheumatologists, the Kenyan scenario is much better than many other countries in Africa [14]. Knowing healthcare workers’ understanding, attitudes and perceptions towards paediatric rheumatic diseases is key to design and implement effective interventions to scale up paediatric rheumatology skills among other healthcare workers [26]. We thus set out to describe the knowledge, attitudes, and practices of healthcare workers towards paediatric rheumatic diseases in the Republic of Kenya.

## Methods

Our study incorporated the COnsolidated criteria for REporting Qualitative studies (Additional file 1 Appendix 1).

This was a qualitative descriptive study of 12 focus group discussions (FGDs) conducted between September and November 2021 among 68 healthcare workers (HCWs) drawn from clinical officers, nurses, general practitioners, and paediatricians. Participants were recruited from across the Kenyan republic through the six regional branches of the Kenya Paediatric Association (KPA) namely Nairobi, North Rift, South Rift, Central, Coast and the Lake Region.

Each cadre of healthcare worker had 3 focus group discussions (FGDs) conducted with five to eight participants. The primary investigator, a paediatric rheumatologist (AM) in Kenya was trained by and worked with experienced Kenyan qualitative researchers (PM and AL) throughout each stage of the FGDs.

Participants were recruited purposively by the study principal investigator-PI (AM) using a two-stage sampling technique. The first phase was purposive sampling [27] where the PI contacted HCWs known to her through KPA to ascertain who was interested in participation. The second phase was snowball sampling [28] where the initial recruited participants were requested to recommend at least 3 members from each healthcare cadre of interest to participate. The proposed participants were then contacted by the study assistant to ascertain their willingness to participate. Participants were eligible for enrolment if they were aged 18 years or older and licensed to practice. Since English is the official language of instruction in Kenya’s HCW training, language was not a deterrent to participation. Participants were excluded if they were unable to dedicate 45–60 minutes of their time for the FGDs, or submitted incomplete consent forms. Participants who were eligible for the study then selected their preferred dates and time for participation in the FGDs. This then was used by the study investigators to constitute and schedule the various FGDs.

A moderator’s manual was prepared, and pilot tested to ensure appropriateness as an FGD guide. Active consenting process was followed. Information sheets (Additional file 2 Appendix 2) and consent forms (Appendix 3) were emailed to those participating in the interviews in advance and sent back via email to the investigating team upon completion. The focus groups were conducted in English via Zoom Communications (copyright 2021) between September and November 2021. Each was recorded using the Zoom record functionality. Eight of the FGDs were conducted by AM while PM conducted the remaining four.

The data was anonymized and transcribed per verbatim. Notes of the focus group proceedings were used to cross-check for consistency. All forms, recordings and transcripts were managed according to ethical guidelines.

### Data analysis

A reflective thematic analysis was conducted using NVIVO 12 software [29]. Data familiarization was done by going through each quote by participants to deduce the key message. Initial coding where themes and sub-themes were grouped into categories was done by AM and PM. A social scientist (AL) conducted a separate coding and analysis of a sub-set of transcripts. This followed a series of iterative meetings among the three researchers where linkages in themes were identified, codes were merged into categories and themes. In the final stage of the data analysis, we conducted a “member checking’ [30] process to check the accuracy of our findings. This involved sharing our findings with some study participants from each cadre, to ascertain if our findings were a reflection of their actual views and experiences.

### Ethical considerations

Ethical approval was obtained from the AKU-EA Institutional Research Ethics Committee (Ref 2021/IERC-50(v2)) and the Kenya National Commission for Science, Technology and Innovation (NACOSTI) research permit (NACOSTI/P/21/11789). All participants provided written informed consent to participate in the study.

## Results

### Participants

Very few of those invited (1 paediatrician and 3 general practitioners) declined to participate in the study due to lack of time and paucity in interaction with paediatric patients respectively. Among the 68 participants, 75% (53 of 68) were female and the mean age was 36 years (range 31–40 years). Fifty percent of the respondents (34 of 68) work in the public sector. Participants were enrolled across 20 different counties in 6 regions namely Nairobi region (*n* = 12; 18%), Lake Region (*n* = 16; 24%), North Rift region (*n* = 7; 10%), South Rift region (*n* = 12; 18%), Coast region (*n* = 8; 12%) and Central region (*n* = 13; 19%). Participants included 11 (16%) clinical officers, 12 (18%) nurses, 10 (15%) general practitioners, 27 (40%) paediatricians and 7 (10%) others (research nurse, emergency physician, family physician, general physician, sleep coach, cardiac technologist, and neonatologist). Among the participants, 17 (25%) were diploma holders, 26 (38%) had a Bachelor’s degree and 24 (35.3%) had a Master’s degree in their respective fields of medicine.

Our study unveiled 3 themes;

1. HCWs have a variable understanding of paediatric rheumatic diseases.
2. There is an interplay between lack of knowledge and health system factors which contributes to late diagnoses and management of paediatric rheumatic diseases.
3. The complexity and chronicity of paediatric rheumatic diseases influence HCWs’ attitudes

1. **Varied understanding of paediatric rheumatic diseases**

The first theme emerging from our analyses indicates a varied understanding of paediatric rheumatic diseases. Some participants demonstrated a good understanding of paediatric rheumatic diseases, describing what these diseases are, including symptoms and affected parts of the body. Others reported a lack of knowledge and awareness among healthcare professionals, particularly among non-specialists.

- ***Adequate understanding of the disease by professionals***

In the following quotes, participants show some understanding of paediatric rheumatic diseases:

*What I know is that paediatric rheumatology are basically autoimmune conditions that affect the paediatric patient and most of them are characterized by joint pains. Most of them affect the joints, but they can affect structures or body organs outside the joints and all of them...*

### 50 year old female clinical officer

*I believe they are usually chronic illnesses in children that sometimes will affect their musculoskeletal system like the joints, the long bones, and most of the times it will present with pain and sometimes the pain could be debilitating, they are not able to perform like walking well, playing …*

### 32 year old male clinical officer

*Others are deforming, life-threating together with others that are moderately severe. They do occur in paediatric age but that does not mean it does not occur in the other ages.... Others also have autoimmune components that means the body probably fights its own tissues.*

### 37 year old male general practitioner

(b) ***Limited knowledge among non-health care specialist and the public***

Knowledge about paediatric rheumatic diseases was still described as limited among non-specialists.

*Personally, speaking from a personal experience, I think rheumatological disorders are not well known or widely known more so among non-specialists like us. So you find that there are a lot of gaps and when you get a patient who has a rheumatological disorder, before you get to that diagnosis, you will have done almost everything [other medical tests] …*

### 24 year old male clinical officer

*I think it is a specialty that is unknown to many … not many people know whether paediatric rheumatologists even exist.*

### 27 year old male clinical officer

Participants commented that knowledge was lacking among the public. For example, participants reported how parents and caregivers brought their children when the disease was at an advanced stage, which they attributed to their [parents/caregivers] lack of knowledge. Upon inquiry of the free pmm online course, none of the participants knew about this resource.

*Most of the children ...are always brought by parents at a rather later stage of the disease already out of the parents not knowing .... So, we have not really been able to do much other than pain control and physiotherapy ...*

### 33 year old female clinical officer

*I have never heard of pmmonline.*

### 35 year old female Paediatrician

Our study also found that, due to limited knowledge among the public, some rural communities may resort to using herbal medicine without seeking clinical care, which may delay diagnosis.

*… from the community … .we have had issues with use of herbal medicine especially from mothers-in-laws and all that...I think they have a negative influence towards these children. Many are those who arrive already in renal failures and all that...kidney injuries out of exposure to a lot of herbal intoxication in the name of treatment and in the name of straightening their muscles …*

### 28 year old female clinical officer

2) **Interplay between lack of knowledge and health system factors contributes to late diagnosis and mismanagement**

- **Late diagnosis and poor management**

Data suggests that HCWs’ limited knowledge had a direct bearing on the timely diagnosis of paediatric rheumatic diseases. Symptoms were described as complicated thus leading to the late diagnosis. HCWs insufficient knowledge implied that on many occasions, they confused paediatric rheumatic disease symptoms with infections ultimately resulting in diagnostic delays.

*… and I think because most of the paediatric rheumatological disorders present with a fever, one thing that everybody jumps to is usually sepsis. You think it must be an infection somewhere more so being that the patient is paediatric …*

### 24 year old male clinical officer

*… most of the time our index of suspicion is maybe more focused on infections, so we tend to miss them but if they are detected early before the disease progress, prognosis is usually good.*

### 36 year old male clinical officer

In addition, HCWs were described as lacking knowledge to distinguish between rheumatology and orthopaedic conditions. Similarly, delayed diagnoses may also arise from HCWs assumption that rheumatic diseases affect only older people and not children. The following narratives highlight some of these challenges.

*Initially* [there was] *big confusion between paediatric rheumatology and orthopaedic. Often children were being misdiagnosed with orthopaedic conditions. … There is misunderstanding of these conditions, as we believe that rheumatoid conditions are for the old.*

### 43 year old female nurse

*… We experience a lot of problems you find that you diagnose a child with arthritis, but you find your colleagues challenging you that arthritis is not for children. Those are negative attitudes from our colleagues.*

### 38 year old male clinical officer

*...But from where I sit, there was a time I was suspecting arthritis in a 4-year child but it took the lab guy by surprise that I am sending the child for lab investigations … they will take that such arthritis or bone infections are for the elderly. It is less common in children.*

### 27 year old male clinical officer

Even when paediatric rheumatic disease is correctly diagnosed, some HCWs may not be confident if they got the diagnosis and management correct, implying that chances of mismanagement are high.

*I have seen probably a few cases in the clinic, which I suspect some rheumatological conditions ... sometimes it is not even very clear what exactly we are managing, we would just think about something and then treat it that way and just hope that the patient improves and most of them do improve....*

### 27 year old female general practitioner

Lack of clarity on management was augmented by lack of protocols. HCWs noted that they had never seen a protocol on how to manage these diseases.

*… like everybody is not so sure on what to do since I’ve not seen a protocol, no. When there is a protocol, you know when it’s like this, you do this, this, this, this, but now there is no protocol, everybody is not so sure of the specifics that needs to be done.*

### 48 year old female nurse

Lack of protocols and proper guidelines may make it difficult to follow up children diagnosed with rheumatic diseases in the system as exemplified below.

*… we hardly do follow-up on those children when they go away, so I’m not sure about the prognosis because I would love to know and hear that they are growing well, and that they.*

*live long.*

### 43 year old female nurse

In addition, participants observed limited opportunities such as continuous medical education to improve their knowledge.

*… I would say that it is one of the disciplines whereby I haven’t even heard of a CME (continuing medical education) of what is to be discussed about paediatric rheumatology and therefore it is one of the forgotten disciplines.*

### 30 year old female general practitioner

(b) **Health system factors a barrier to early diagnosis**

(i). ***Lack of clinical pathways***

The lack of knowledge among healthcare workers interplays with lack of clear clinical pathways, implying that many children with paediatric rheumatic disease may initially be treated for other diseases common in Africa, like malaria and other infectious diseases. Furthermore, this is amplified by the lack of clinical care pathways in the current Kenyan health system, which results in late diagnosis and poor management. HCWs are not adequately equipped with the right knowledge, and hospitals lack the right tools and standard operating procedures, impeding timely diagnoses.

*It’s something that is not so common though … usually, we get these conditions especially referrals from peripheral health facilities, ... the child has been maybe mismanaged, managed for malaria, the child came with joint pains managed for malaria without even testing whether the BS [blood slide for malaria] is positive. So, by the time they come to the bigger hospitals, they are usually a bit late.*

### 50 year old female clinical officer

*I think I would say, the difficulty is in getting the diagnosis right, I feel uncomfortable...I would really feel uncomfortable making a final decision that this is what it is most of the time for these conditions. That’s tough.*

### 27 year old female general practitioner

(ii). ***Children pushed along in the system to other physicians, due to lack of clinical care pathway***

Our information from participants suggests that lack of clinical care pathways may have a far-reaching consequence for children with rheumatic diseases. It seems clear that the current health care system does not apportion responsibility and accountability, leaving the children vulnerable in the hands of inadequately trained health care professionals who ‘push’ them in the system without making decisions.

*I would say, as well for my colleagues, it’s that tough. And just like my colleague xxxx said that if you have a rheumatologist that you know, you just kinda (kind of) push them that side so that you don’t have to really think a lot about making that decision of what exactly it is. I think it is such a difficult thing to come to a final diagnosis.*

### 27 year old female general practitioner

*...like the other day I had a suspicion of SLE (systemic lupus erythematosus) and I was like, I have never seen SLE in kids and there was an excitement of this … but I was very quick to just do the baseline investigations and push forward to my seniors.... We would feel like we just need to do the bare minimum...just keep her alive until the seniors are able to attend to her …*

### 29 year old female general practitioner

*… apart from giving them the junior aspirin, … … it’s a scope that belongs to another person or another level. … So whenever I see a child, I always feel like, even if I see her now, I’ll push to another person, beyond my scope.*

### 55 year old female clinical officer

*Then the fact that to reach the diagnosis of maybe rheumatoid arthritis, most of the.*

*time, the patient has gone through so many guess work from different doctors, from different.*

*places by the time they are arriving at paediatric rheumatology clinic, they’ve been mismanaged all over.*

### 53 year old female nurse

3) **Complexity and chronicity of paediatric rheumatic diseases influence HCWs attitudes**

Our data suggest that HCWs’ perceived complexity of the disease influenced their attitudes towards the disease. When HCWs felt disempowered to deal with the disease, they felt frustrated, scared and stressed as seen in the following quotes.

*I think the word I can use is that of frustration. When you are dealing with a condition that you are not really empowered to diagnose and possibly initiate management, the best descriptive word is frustration and then you meet the client that has it because they need care that they ought to get even if you had good ideas …*

### 52 year old male clinical officer

*I find it quite stressful when I handle patients with a rheumatological disorder especially those with very severe joint pains and very young patients. … It’s usually very frustrating especially when the pain is not going away for the parent, the doctor, the nurses, the other healthcare workers because babies will just be screaming in the ward.*

### 35 year old female Paediatrician

*For me I think it’s a scary condition. That’s what I’ve seen. You just told us to say attitude, to me it’s scary. I think it’s a complicated disease that if it’s not handled, things can go south, so its scary disease for me.*

### 58 year old female nurse

Some participants did report having positive attitudes towards paediatric rheumatic diseases. As illuminated in the quotes below. This broke the monotony of seeing patients with contextually common illnesses such as pneumonia and diarrheal diseases. It also caused the healthcare workers to search for new knowledge, an experience which they perceived positively.

*I think I’m one of those people who love a mystery or something that really gets you scratching your head and you find that most of these children will have very non-specific symptoms which will involve a lot of reading, a lot of research looking at what they have and comparing it to other so... I’m actually very fascinated about that because they are conditions that they are very intricate, like you need to be very careful. You need to be very accurate or you need to be very... You look at it very critically and make the best decisions because it involves a very high level of knowledge when it comes to diagnosis and even management, so I actually quite enjoy managing patients who present with rheumatological conditions. It beats the monotony of the everyday pneumonia or diarrheal diseases which are usually very common.*

### 24 year old male clinical officer

Some HCWs saw this as an opportunity to learn something new.

*I would say it’s an interesting subspecialty. There is always something new to learn about it. The other thing, sometimes in terms of, when I make a diagnosis of a rheumatic disease, sometimes, it makes me a little bit kind of not sympathizing but more of empathy towards the patient …*

### 27 year old female general practitioner

Table 1 below summarizes the findings of our study;

**Table 1:** Codes, categories, and themes

Codes		Categories	Themes
**1**	**Description of paediatric rheumatic diseases**	1. 1. 1. 1. Adequate understanding of the disease by professionals	**Variable understanding of paediatric rheumatic diseases**
	○ Described as autoimmune condition		
	○ Affects joints and musculoskeletal system		
	○ Chronic in nature		
	**Professional’s perception of HCWs knowledge levels**	2. 1. 1. 1. Limited knowledge of the disease among HCWs and the public.	
	○ Inadequate knowledge of paediatric rheumatic diseases		
	○ Misunderstanding of rheumatological disorders		
	○ Misunderstanding of age groups affected		
**2**	**HCWs perception towards management**	1. 1. Late diagnosis and poor disease management	**Interplay between lack of knowledge and health system factors contributes to late diagnoses and management**
	○ Variation in symptoms		
	○ Understanding symptoms	2. 1. 1. Health systems factors are a barrier to early diagnosis	
	○ Missed symptoms		
	○ Symptom management		
	○ Unclear referrals	○ Lack of clinical pathways 1.	
	○ Referred on to others HCWs feeling inadequate		
**3**	**Attitudes of HCWs towards PRDs**	1. 1. 1. 1. 1. Both negative and positive attitudes reported.	**Complexity and chronicity of the disease influenced HCW attitudes**
	○ Healthcare workers felt frustrated		
	○ Healthcare workers felt frightened and immobilized		
	○ Paediatric rheumatic diseases are complex, uncommon, not well known		
	○ Complexity of the diseases leads HCW to look up answers		

## Discussion

Our study revealed varied understanding of paediatric rheumatic diseases among HCWs in Kenya. The complexity, chronicity of paediatric rheumatic diseases, and interplay between lack of knowledge and health system factors, all impacted on the perceptions of healthcare workers towards paediatric rheumatic diseases. Limited experience, lack of training in paediatric rheumatology by healthcare workers, further impacts negatively on their perceptions towards paediatric rheumatic diseases. Some of these negative consequences were also highlighted in a review by Lewandowski [15].

Rheumatic diseases are often difficult to understand and our study participants highlighted their lack of knowledge and uncertainty in managing paediatric rheumatic patients [28]. Our findings are congruent with the work of Jandial et al. who highlighted a lack of confidence among general practitioners in paediatric musculoskeletal (MSK) clinical skills in United Kingdom [31]. Educational needs for healthcare practitioners vary and should be tailored in accordance with individual characteristics such as gender, age, educational background and social context [28].

Paediatric rheumatology education is still sub-optimal in many regions across the globe [31, 32]. Trainees in primary care have previously reported that training in paediatric MSK assessments is inadequate, leading to poor performance [27]. Furthermore, exposure to paediatric rheumatology training is limited to centres with paediatric rheumatologists [27]. Formal instruction in paediatric MSK assessment within paediatric residency programs [27] or undergraduate programs is limited [27].

Some strategies that may be employed to bridge the gap in knowledge include mass education of the populace in different languages across various media platforms. Other strategies to consider include availing routine information in healthcare facilities and educating community health workers who would then act as ambassadors within the community for prompt identification and early referral of paediatric rheumatic patients.

The interplay between lack of knowledge and health system factors has an impact on the inconsistent practices and clinical care offered which adversely influences patient outcomes [33]. Our study revealed that frustrations experienced by healthcare workers (knowing that the care they provide is sub-optimal) leads them to refer patients to other health care providers. Barber and colleagues similarly found that inadequately treated paediatric rheumatic patients leads to negative psychological impacts on patients and healthcare workers [34]. This state of helplessness puts the healthcare worker at risk of burnout, which would further compromise care to the patient. Strategies to help empower HCWs include mass education and sensitisation. Screening tools to help them accurately detect suspected cases may help minimize their feeling of inadequacy.

Health care access is a complex issue with many contributing factors [35]. Availability and accessibility are just part of a bigger picture that evidently affect paediatric rheumatology referral decisions among HCWs [35]. Due to limited resources, we must question whether improving care for paediatric rheumatic patients should focus on expanding the reach of care or enriching current care models [35]. As clinical care models are implemented, further research is needed to better objectify the opportunity cost [35].

Our study demonstrated that many healthcare workers often treat paediatric rheumatic patients for infections given that they share similar clinical features such as fever. In her review, Lewandowski reiterated this challenge of differentiating rheumatic disease from infection [15]. Diseases, such as systemic JIA and systemic lupus erythematosus (SLE) may be managed as malaria or Human Immunodeficiency Viral (HIV) disease, given the overlap of presenting symptoms [15]. Matsumoto and co-workers highlighted other challenges that our participants also face in their clinical practice, such as inability to perform a physical exam, the perceived high cost to institutions for management, and time constraints [36]. As a result, some of our participants admitted to “pushing” patients to other health care providers, to avoid dealing with these challenges.

Increased recognition of rheumatic disease by HCWs is essential for addressing delayed diagnosis [15]. Exposure to MSK training during undergraduate and postgraduate training will help bolster confidence in assessing rheumatic diseases and improve timely referral and diagnosis [15]. The local HCWs recognition of rheumatic disease should be accompanied by access to diagnostics, and prompt referral to a rheumatologist as needed [15]. In addition, training of future generations of paediatric rheumatologists is necessary to close workforce gaps [15].

In our study, nurse participants expressed that pain control was an important role for them. Rheumatology nurses play a fundamental role in the care of rheumatology patients [37]. A study in United Kingdom (UK) involving 200 nurses revealed drug monitoring, education, counselling of patients, ordering investigations, drug administration and research are performed by nurses [37]. The role of rheumatology nurses has expanded over recent years and includes running specialist clinics, patient education, patient assessment and treatment [15]. The role of the rheumatology nurse is likely to expand soon to include medication prescription [15]. Factors that could enhance their role include: attendance at postgraduate courses; obtaining further qualifications; active participation in the delivery of medical education; training in practical procedures; protected time and resources for audit and research; formal training in counselling; and implementation of nurse prescribing [38].

The practice of rheumatology requires advocacy and education of the public; development, sensitization and implementation of diagnostic and management algorithms, strengthening of health systems to support definitive, and supportive care for paediatric rheumatology patients in order to have optimum patient outcomes [39].

Due to rising awareness of the complexity and chronicity of paediatric rheumatic diseases, there is a greater demand for better health care [40], providing further impetus to improve the paediatric rheumatology care in Kenya. The large patient pool available in Kenya is an untapped clinical resource that could provide the basis for teaching and patient-oriented research. International collaborations with established paediatric rheumatology centres across the globe are needed to develop world-class centres of excellence in disease management and research in Kenya.

One way to address these needs is to introduce modules on paediatric rheumatology in undergraduate and postgraduate training, so that non-rheumatologists can recognize and manage common paediatric rheumatic problems and refer children to specialists early. Kenya is a hub for information technology, and the government’s desire to link all medical colleges by a telemedicine network could provide an opportunity to disseminate knowledge across this vast country, even with limited trained manpower. Paediatric rheumatology in Kenya is at a crucial juncture where, if opportunities are maximized, could lead to significantly better care for children with rheumatic diseases in the region and across the African continent.

### Limitations

Some participants had poor internet connectivity, which led to interrupted transmission of their contributions to the discussion. Also, due to the nature of their work, some participants were not able to stay to the end of the virtual focus groups or log on in time due to emergency cases that they had to attend. However, we had a relatively large number of participants and focus groups, and the content of the discussions was fairly homogeneous. Thus we are relatively confident that our research covered the major relevant themes.

Very few of those invited (1 paediatrician and 3 general practitioners) declined to participate in the study, which is a strength. Regardless, there may have been under-representation of certain cadres of healthcare workers from some regions. Regarding conduct of the FGDs, we tried to minimize bias as much as we could by using standardized procedures and the same moderators/co-moderators for all focus groups. 

### Future directions

Our study has laid the foundation for a qualitative formative work to better understand HCWs perceptions towards paediatric rheumatic diseases in Kenya. Moving forward, we need a better in-depth understanding across the country. This may entail a larger survey in rural and urban areas piloted among community health workers treating those who are marginalized.

It is also equally important to engage with key stakeholders in the health sector on how to best integrate clinical care for pediatric rheumatology patients. The use of ethnographic methods can be explored to analyze the processes of transfer, treatment and follow up of paediatric rheumatology patients. These strategies are proposed in order to help strengthen the health system to cope with the demands of paediatric rheumatology patients.

In conclusion, we are the first to explore the knowledge, attitudes and practices of healthcare workers in Kenya, regarding paediatric rheumatology. Our work forms a foundation upon which hopefully an intervention can be built to meet the educational and clinical needs of health care workers, their patients, and affected families.

## Supplementary Information

**Additional file 1: Appendix 1.** Consolidated criteria for reporting qualitative studies (coreq): 32-item checklist.

**Additional file 2: Appendix 2.** Moderator’s manual for focus group discussions.
