# Supplementary material for: An iceberg I can’t handle: a qualitative inquiry on perceptions towards paediatric rheumatology among healthcare workers in Kenya
Source: Pediatr Rheumatol Online J. 2023 Jan 21;21:6. doi: 10.1186/s12969-023-00790-2 (PMC9862847; doi:10.1186/s12969-023-00790-2)
Supplement: Supplementary file 2 — Additional file 2: Appendix 2. Moderator’s manual for focus group discussions. [file 12969_2023_790_MOESM2_ESM.docx]

## Appendix 2: Moderator’s Manual for Focus Group Discussions

Thank you for accepting to participate a focus group discussion. Running an effective group discussion is a skill and requires planning. This note is intended to assist you in organizing and conducting the group discussions with non-specialist healthcare providers to determine their knowledge, attitudes and practices towards paediatric rheumatology and **pmm, an online paediatric rheumatology learning resource**. It is critical that the voices and perspectives of the paediatricians be captured and reflected. If you have any questions on the guidance, please do not hesitate to contact us (contact information at end of note). We thank you for being a part of this important work!

**I. What are we asking you to do?**

We are asking you to participate in a focused group discussions with non-specialist healthcare workers from different regions of Kenya to better ascertain their educational needs as pertains to paediatric rheumatology.

To make it easier for you to record and share with us your group discussions, we offer this Word file with suggested questions and space to summarize responses. You can e-mail it back to the principal investigator at **angela.migowa@aku.edu**. We hope you will find the questions pertinent, and we encourage you to guide the group to address them.

**II. General principles of guided group discussions**

***Voluntary participation***

Participants must agree to participate in the discussion of their own free will. It is essential that participants understand that their participation is voluntary, no quotes are attributed to any specific participant, and there are no consequences to answering specific questions.

***Confidentiality***

The group organizers and participants must agree to keep confidential any sensitive personal information that is revealed during discussion, unless the individual in question and the other respondents agree that it is required as part of the feedback. The focus group discussion is not an individual survey about facts and numbers or to determine a majority opinion; instead it aims to bring out a rich experience-based analysis through interaction and a range of views (some common, some divergent).

***Participants***

Non-specialist healthcare providers from across the various regions of Kenya representing the 6 regional branches of the Kenyan Paediatric Association i.e Nairobi Branch, Central Branch, North Rift Branch, South Rift Branch, Coastal Branch and Lake Region Branch will be selected using purposeful sampling.

***Participant Consent***

Participants will sign a consent form to participate in the focus group discussion. One copy of the informed consent form should be given to participants and a second copy should be kept by the group facilitator. Participants should be informed if any audio-taping will be used for data collection.

***Demographic data***

It is important to collect anonymous demographic data from group participants. Simple questionnaires for this purpose will be handed out as participants arrive, then collected at the end of the focus group and kept with the tapes of the focus group.

**II. How you should organize the guided group discussion?**

***Select the participants***

Participants will be selected through purposeful sampling using the **Kenya Paediatric Regional Branch Chairpersons’** database. Be sure that participants are able to address the topics raised in the discussion questions and well positioned to provide valuable insight. The group participants should represent the diversity of the larger group about whom we want to learn—for example both men and women, older and younger should have a chance to participate. A convenient time of day and comfortable location for the participants that is responsive to their life circumstances will be selected.

***Invite the participants***

Once the date and venue is set, participants will be contacted via phone and email to re-confirm their participation and arrange for an appropriate date and time for the virtual focus group discussion.

***Record the discussion***

A dedicated note-taker will be assigned to write and type the contents of the discussion. The discussion will be recorded using both audio recording equipment and the note-taker’s notes (which can be refined later with the help of the audio recording). The hand-written notes should be extensive and accurately reflect the content of the discussion, as well as any salient observations of non-verbal behaviour, such as facial expressions, hand movements, group dynamics, etc. A third assistant will be assigned to monitor the audio recording equipment and keep track of time.

***Discussion guides***

The discussion guide is a template but participants will be encouraged to explore the topics in depth, to reflect, to raise their own issues, etc.

***Data collection***

The discussions can be audio-taped if agreed by participants, and transcribed verbatim for analysis. The recordings need to be securely stored until transcribed and then destroyed. The transcription shall not contain information that would allow individuals to be linked to specific statements. Confidentiality will be strictly preserved, except where disclosure is mandated by a court of law.

**III: Tips for conducting focus group discussions**

***Explain the principles of focus group discussions***

Make sure participants understand their rights and express their verbal consent and assure them that their identities and individual statements will not be recorded or published.

***Establish rapport***

The facilitator should outline the purpose and format of the discussion at the beginning of the session to set the group at ease. Participants should be told that the discussion is informal, everyone is invited to speak up, and divergent views and debate are welcome.

Initiating each topic with a carefully crafted question will help participants share their experiences in a focused and meaningful manner. Use judgment in allowing the conversation go its own way for some periods, as long as it’s producing useful inputs per the general thrust of the questions.

***How to keep the conversation flowing***

A few suggested techniques:

• Repeat the question – repetition gives more time to think.

• Pause for the answer – a thoughtful nod or expectant look can convey that you want a fuller answer.

• Repeat the reply – hearing it again sometimes stimulates conversation

• Ask when, what, where, which, and how questions – they provoke more detailed information Use neutral comments – “Anything else?” Ensure that all group members participate In focus groups, it is not uncommon for a few individuals to dominate the discussion.

Sometimes in mixed gender groups, one gender may tend to speak more than the other. To balance participation, and ensure that every participant has an opportunity to contribute to the discussion, one might consider the following strategies:

• Address questions to individuals who are reluctant to talk

• Give nonverbal cues (look in another direction or stop taking notes when an individual talks for an extended period)

• Intervene, politely summarize the point, then refocus the discussion. Minimize pressure to conform to a dominant view point

***Feedback and getting involved***

Participants may be interested to know more about what will be done with the information they provided and/or how other groups in the same or different countries responded. The analysis of people’s inputs will appear in the final report which will eventually be published and made available for access.

***Interview Guiding Points***

1. Experience with paediatric rheumatology (generally)

2. What do you know about PMM online

3. Any challenges accessing PMM online

4. What do you know about about paediatric rheumatic diseases (PRD)

5. Knowledge of guidelines and protocols for diagnosing and managing PRD

6. Sources of knowledge for paediatric rheumatology

7. Attitudes of a) respondents and b) peers towards paediatric rheumatology

8. Attitude towards Paediatric Rheumatology

9. How can you influence a) peers and b) health facility to improve the care accorded to paediatric rheumatic patients

10. Community representatives who help make decisions at the paediatric clinic. List them, are they able to influence decisions, why or why not.

E.g. CHVs, they help make decisions because they are trained in community health prevention and promotion.

11. Roles played in management of paediatric rheumatic conditions

12. Whether role was successfully handled or not

13. Proposals to improve practice of paediatric rheumatology

14. Challenges faced in practicing paediatric rheumatology

15. Most important issues that should be incorporated into pmm to improve KAPs of paediatric rheumatology at the local health facility

_____One Key Word for the Online App that makes the biggest difference to PRD Patients

~~~~~~~~~~~~~~~~~~~~~~~~~~~~~~~~~~End~~~~~~~~~~~~~~~~~~~~~~~~~~~~~~~~~~
